# The potential links between human gut microbiota and cardiovascular health and disease - is there a gut-cardiovascular axis?

**DOI:** 10.3389/fgstr.2023.1235126

**Published:** 2023-10-16

**Authors:** Cátia Almeida, J. Guilherme Gonçalves-Nobre, Diogo Alpuim Costa, Pedro Barata

**Affiliations:** ^1^ Biomedicine Department, Biochemistry Unit, Faculdade de Medicina da Universidade do Porto (FMUP), Porto, Portugal; ^2^ Faculdade de Ciências da Saúde (FCS-UFP), Universidade Fernando Pessoa, Porto, Portugal; ^3^ Hospital Garcia de Orta (HGO), E.P.E., Almada, Portugal; ^4^ Instituto de Saúde Ambiental (ISAMB), Faculdade de Medicina da Universidade de Lisboa (FMUL), Lisboa, Portugal; ^5^ Instituto de Medicina Preventiva & Saúde Pública (IMP&SP), Faculdade de Medicina da Universidade de Lisboa (FMUL), Lisboa, Portugal; ^6^ PTSurg – Portuguese Surgical Research Collaborative, Lisboa, Portugal; ^7^ Medical Oncology Department, Hospital de Cascais Dr. José de Almeida, Cascais, Portugal; ^8^ Haematology and Oncology Department, CUF Oncologia, Lisbon, Portugal; ^9^ NOVA Medical School, Faculdade de Ciências Médicas, Lisbon, Portugal; ^10^ Instituto de Investigação e Inovação em Saúde (i3S), Universidade do Porto, Porto, Portugal; ^11^ Pathology Department, Centro Hospitalar Universitário do Porto (CHUP), Porto, Portugal

**Keywords:** gut microbiota, gut-heart axis, dysbiosis, cardiovascular diseases, atherosclerosis, hypertension, TMAO, SCFAs

## Abstract

The gut-heart axis is an emerging concept highlighting the crucial link between gut microbiota and cardiovascular diseases (CVDs). Recent studies have demonstrated that gut microbiota is pivotal in regulating host metabolism, inflammation, and immune function, critical drivers of CVD pathophysiology. Despite a strong link between gut microbiota and CVDs, this ecosystem’s complexity still needs to be fully understood. The short-chain fatty acids, trimethylamine N-oxide, bile acids, and polyamines are directly or indirectly involved in the development and prognosis of CVDs. This review explores the relationship between gut microbiota metabolites and CVDs, focusing on atherosclerosis and hypertension, and analyzes personalized microbiota-based modulation interventions, such as physical activity, diet, probiotics, prebiotics, and fecal microbiota transplantation, as a promising strategy for CVD prevention and treatment.

## 1 Introduction

Humans have a diverse and dense ecosystem of microorganisms called the human microbiota, which has been known for almost a century. However, we are only now starting to grasp many of these microorganisms’ functions in human health and development (1).

The human microbiota comprises more than 100 trillion microbial species, within which bacteria, fungi, viruses, and protozoa are distinguished (1, 2). These microorganisms, together with their genes (microbiome), form a dynamic microbial community that inhabits different areas of the human body, playing a vital role in the host’s health (1). The site of the human body that hosts the most significant number and diversity of microorganisms is the gastrointestinal tract, more precisely the gut, having a significant impact on human homeostatic processes such as nutrient metabolism, maintenance of intestinal mucosal barrier integrity, regulation of satiety, defense against pathogens either by pH modification or secretions of antimicrobial peptides or changes in cell signaling pathways, and development of the immune system (3, 4). These microorganisms coexist in harmony with their host, demonstrating a symbiotic relationship. Although a balance between the microbiota and its host must be observed to optimize metabolic and immunological functions, there is no ideal composition because each person has a unique microbiota (5). Thus, considering gut microbiota characteristics such as high diversity, stability, and resilience, and the symbiotic interactions with the host, we can define it as a superorganism (6, 7). Firmicutes, Bacteroidetes, Proteobacteria, and Actinobacteria, are the four major phyla in the gut microbiota, and in healthy adult individuals, the first two prevail (8, 9). Its composition remains stable over time, but the microbiota is characterized by some volatility, demonstrated by a diverse set of genetic and environmental factors like dietary composition, social interactions, infections, and antibiotic exposure, that can shape its composition (10, 11).

Most studies prove that the balance between the microbial species in the gut microbiota is fundamental for maintaining the body’s homeostasis (11). The term dysbiosis refers to an imbalance in the microbiota composition with a consequent change in its functions, whereby its normal beneficial state changes to a possible harmful state for human health, with pro-inflammatory effects and immune dysregulation associated with several disorders (12). Increasing evidence points to the possibility of using variations in the F/B ratio, the ratio of the microbial communities Firmicutes and Bacteroidetes, as a biomarker for pathological disorders (13). However, a growing body of proof suggests that gut microbiota impacts intestinal disorders and numerous extra-intestinal disorders such as neurological disorders, cardiovascular diseases (CVDs), cancer, and many others (14). Understanding the cause or consequence of this situation and how to maintain or restore the composition of the gut microbiota will be very helpful in developing new therapeutic interventions (15).

In the past decade, CVDs have emerged as the leading cause of death worldwide, taking an estimated 17.9 million lives yearly (16, 17). Besides genetic factors, environmental factors and intestinal microbiota were also acknowledged as one of the main factors for the development of CVD. Also, diabetes, obesity, and metabolic syndrome, three major risk factors for CVDs, have been linked to intestinal dysbiosis as a risk factor for development (18, 19). One example of the potential link between gut microbiota and CVD is the production of trimethylamine N-oxide (TMAO), a compound that has been linked to CVD, with high plasma TMAO levels having a close association with the risk of developing atherosclerosis (16, 19, 20).

The purpose of the present review is to explore the role of the gut microbiota concerning the development of CVD, focusing on our previous works (20), and the most current evidence regarding TMAO as a biomarker for CVD and the effects of its precursors, choline, and carnitine, on TMAO formation and the associated high CVD risk, as well as the beneficial effects of short-chain fatty acids, bile acids and polyamines in CVD development (21–23).

## 2 Gut microbiota ecology and its implication on cardiovascular diseases

### 2.1 Bacterial microbiota

Emerging evidence suggests that the gut microbiota may be an essential contributor to the development of CVDs, such as atherosclerosis, hypertension, coronary artery disease, and stroke. Many researchers have reported a connection between CVD phenotypes and changes in the relative abundance of specific microbial taxa or the richness or variety of the bacteria in the gut (24, 25).

The gut barrier is a complex system that separates the intestinal lumen from the rest of the body (26). It plays a critical role in maintaining the health and integrity of the body by preventing the translocation of harmful substances and microorganisms from the gut into the rest of the systemic circulation (26, 27). In a healthy individual, the intestinal barrier is intact and functions appropriately, being maintained by physical factors like tight junctions between epithelial cells, mucus production, and mucosal immunity (28, 29). The barrier comprises several layers, including the mucus layer, the epithelial cell layer, and the underlying immune system (26, 30). The mucus layer is a thin layer that coats the gut’s surface and acts as a physical barrier to prevent the adherence of pathogens and harmful substances. The epithelial cell layer is composed of a single layer of cells that forms the outermost layer of the gut and acts as a selective barrier allowing the passage of nutrients and water into the body while preventing the translocation of harmful substances. The underlying immune system also plays a role in maintaining the integrity of the intestinal barrier, helping to prevent the invasion of pathogens and other harmful substances by producing antibodies and other immune cells that can target and neutralize them (26, 27). According to the leaky gut theory, decreased gut barrier function has been linked to health problems, causing bacterial compounds to enter the bloodstream of the host, which causes an inflammatory response (31). Numerous studies demonstrate altered intestinal integrity in heart failure patients, and higher blood levels of pro-inflammatory cytokines are associated with more severe symptoms and worse outcomes. These situations have also been reported in conditions like inflammatory bowel disease, food allergies, autoimmune disorders, and CVD (31, 32).

Some evidence suggests that lipopolysaccharides (LPS) and leaky gut may be related (33). The LPS are large molecules found in the outer membrane of gram-negative bacteria, also known as endotoxins. They are released when gram-negative bacteria die and lyse, releasing their content into the surrounding environment. Therefore, LPS can cause acute and chronic inflammatory reactions when they enter the bloodstream, as the immune system recognizes them as foreign invaders and mounts a range of physiological responses with toll-like receptors (TLR)-4 being the key interlocutor and determine cytokine cascade and caspase activation (33, 34). Recently, studies have been shown to increase intestinal permeability in animals, with some describing that individuals with leaky gut have higher levels of LPS in their bloodstream and are more predisposed to developing CVD (35, 36). However, the relationship between LPS and leaky gut still needs to be fully understood, and more research is needed to confirm these findings. Another example is the pathogenic gram-negative bacteria *Salmonella* spp. which can breach the intestinal epithelium and alter tight junctions, causing diarrhea via water and electrolyte loss into the intestinal lumen (26). There, inflammation brought on by bacterial translocation to the gut mucosa because of gastroenteritis might worsen gut barrier failure and create a vicious cycle (37, 38).

One way the bacteria from gut microbiota may affect cardiovascular health is through its impact on inflammation (39). Inflammation is a normal immune response to injury or infection; however, chronic low-grade inflammation is a critical factor in the development of CVDs, and gut microbiota dysbiosis has been shown to lead to inflammation through the production of various signaling molecules and the activation of immune cells (40). This may be due to certain types of bacteria that can produce substances that can stimulate an inflammatory response, like pro-inflammatory cytokines that can stimulate acute-phase reactants and contribute to atherosclerosis (41). In addition, the gut microbiota may also influence CVD through its effects on metabolism by affecting lipids and glucose and leading to dyslipidemia and insulin resistance, known risk factors for CVD (39). There is also evidence that the gut microbiota may be involved in developing arterial stiffness, a key predictor of CVD. This may be due to the influence of the gut microbiota on the production of SCFAs, which have been shown to affect arterial stiffness in animal models (42).

Altogether, the evidence suggests that bacteria from the gut microbiota play a significant role in the development of CVDs. However, further research is needed to understand the mechanisms underlying this relationship and how this information can prevent or treat CVD.

### 2.2 Viral microbiota

The viral microbiota refers to the DNA and RNA viruses, including eukaryotic viruses, bacteriophages, retroviruses and archaeal viruses, living in and on the human body, which are highly heterogeneous across populations (43, 44). These viruses can significantly impact the overall microbiota (44). Phages are classified as either lytic or lysogenic; lytic phages reproduce by infecting and killing their host cells, while lysogenic phages integrate their genetic material into the host cell’s genome and replicate. Some phages are thought to have a symbiotic relationship with their host cells, while others may cause harm (45). The virome is a relatively new area of research, and much is still unknown about the types and roles of phages in the human body. It is thought to be highly diverse, with thousands of different types of phages present in the body. Recent studies have characterized the virome at several body sites, including the skin, mouth, gut, and respiratory tract. Some phages are thought to play a role in maintaining the microbiota’s balance and protecting against infection by harmful bacteria (46).

There is evidence suggesting that the viral microbiota may be related to CVD. Some studies have found that individuals with CVD have a different virome profile than those without and that specific phages may be associated with an increased risk of CVD. De Jonge PA et al. study has provided us with the knowledge that viromes from individuals with metabolic syndrome, a well-known risk factor for CVD, have less richness and relative abundance than those belonging to healthy controls (47). This study identified increased viral clusters associated with Bacteroidaceae in the metabolic syndrome population. Moreover, Bacteroides prophages may influence bacterial metabolism, hence modifying microbiota composition in the gut. Additionally, the authors discovered a potential new viral biomarker of metabolic syndrome, VC_818_0, a phage from Roseburia/Blautia bacteria belonging to the Candidatus Heliusviridae phage family. Since the abovementioned bacteria are usually found in healthy microbiota compositions, VC_818_0 phage, which contains genes with metabolic expression, may change the metabolic behavior of these bacteria (already described in marine environments) (48, 49), promoting a deleterious modification of their virulence, hence, enhancing metabolic syndrome (47).

Furthermore, evidence sheds light on the effect of the Microviridae family on coronary heart disease (CHD). First, it was observed that CHD patients had an increased quantity of Virgaviridae and lower amounts of enteric viruses than healthy controls, perhaps due to the type of diet or even the medical therapy (50). Afterward, it was noted that the virome from normal gut individuals was dominated by phages from Siphoviridae, Podoviridae, and Myoviridae with lower quantities of Microviridae. In contrast, CHD viromes are mainly dominated by Microviridae and Virgaviridae, with fewer Siphoviridae, Podoviridae, and Myoviridae (51, 52). So, this study found no causal correlation between CHD patients and their viromes (53).

### 2.3 Fungi microbiota

Fungi are a diverse group of microorganisms found in various body sites. Like bacteria, fungi are an essential part of the microbiota and play multiple roles in human health. Several fungi types are considered standard parts of the human microbiota, including yeasts, such as Candida and molds (54). These fungi are typically harmless when present in small amounts, but when they grow out of control, they can have harmful effects on human health. For example, some fungi in the gut produce enzymes that help to break down food, while others may have a role in regulating the immune system (55). Some evidence suggests that the mycobiome may be related to CVD, a condition affecting the heart and blood vessels. One example is Candida, which is more prevalent in individuals with CVD than those without (56, 57). Candida has been shown to produce toxins that can damage blood vessels and promote inflammation, which may contribute to the development of CVD (57). Other fungi, such as Aspergillus, have also been linked to an increased risk of CVD (58).

The CVD does not happen randomly. Indeed, some risk factors are already identified, such as atherosclerosis, and hypertension, among others (19, 59). With that being said, a new study explored the role of mycobiome in the physiopathology of the abovementioned risk factors. Atherosclerosis is a significant risk factor involved in CVD, extensively analyzed, and is related to the acute and chronic expression of CVD. It was demonstrated that some fungus species might be correlated with atherosclerosis. Mucor spp., from the family Mucoraceae and phylum Zygomycota, is associated with decreased carotid intima-media thickness (cIMT). Moreover, individuals with obesity, when the mentioned fungus is detected, had the same risk as non-obese individuals. With further exploration of Mucor spp., it was possible to demonstrate that Mucor racemosus can be used as a cardiovascular risk biomarker since it was related to a decreased risk on Framingham Risk Score and cIMT (60).

A more relevant risk factor for CVD is hypertension. Mycobiome has a relevant influence on hypertension development. It was observed that individuals in a state of pre-hypertension share the same bacterial and fungal microbiota modifications as individuals with diagnosed hypertension. Interestingly, bacterial richness and diversity reduce when an unhealthy state is reached, while fungal diversity is increased precisely when a pathology, like hypertension, is present. Moreover, some fungi can be used as potential biomarkers for hypertension, such as the increased quantity of Malassezia spp., which is known to promote pro-inflammatory states, as well as diminished concentrations of Mortierella, which can be found in healthy individuals, with an apparent probiotic effect in the bacterial species (61). However, the relationship between the fungi microbiota and CVD still needs to be fully understood; more research is required in order to confirm these findings and determine the exact role of fungi in the development and progression of CVD. In the meantime, maintaining a healthy lifestyle, including following a healthy diet and regular exercise, is crucial to reducing the risk of CVD and other health problems.

## 3 Gut microbiota metabolites

Gut microbiota can modulate human metabolism by producing small molecules, such as the transformation of dietary components into hormone-like signals or physiologically active metabolites, that play vital roles in inflammatory signaling and interact directly and indirectly with host immune cells. These metabolites can have a variety of effects on the body, both positive and negative (62). Some metabolites, such as SCFAs, have been shown to have several beneficial effects on the body, while others, like TMAO, have been linked to an increased risk of certain diseases (63, 64). The role of gut microbiota metabolites in health and disease is an active area of research, but it still needs to be fully understood how these metabolites influence the body. However, understanding the role of gut microbiota metabolites may help researchers develop strategies to prevent or treat several conditions.

### 3.1 Short chain fatty acids

The SCFAs are carboxylic acids with less than six carbons, produced by the fermentation of dietary fibers and non-digestible carbohydrates, that evade digestion by host enzymes in the upper gut and are metabolized by bacteria in the cecum and colon, with decline concentrations from proximal to the distal colon as the substrates used for fermentation are exhausted gradually (63). These compounds are essential for maintaining gut health and have been shown to have several beneficial effects on the body, including reducing inflammation, improving insulin sensitivity, regulation of gene expression, and regulating the immune system (62, 65). Diet composition directly influences the production of SCFAs; specifically, Bacteroidetes and Firmicutes can ferment indigestible fibers in the gut to produce acetate, propionate, and butyrate, respectively, that can be absorbed and used as an energy source (63, 66).

Acetate is the most abundant SCFA and is thought to have diverse beneficial effects on the body, including reducing inflammation, preventing the overgrowth of harmful bacteria, regulating pH, and improving gut barrier function. Propionate is also thought to have anti-inflammatory effects, limiting the growth of dangerous bacteria. It has been shown to improve insulin sensitivity in animal studies, which may be beneficial for people with diabetes or at risk of developing diabetes. Moreover, its potential role in appetite control has been studied, suggesting that propionate may help reduce food intake and promote weight loss, affecting the release of hormones involved in appetite regulation, like ghrelin (67). Butyrate is considered the primary energy source for colonic epithelial cells, and its deficiency has been associated with the development of colitis and cancer (68). Furthermore, it plays a role in maintaining gut barrier integrity by strengthening the tight junctions between epithelial cells that control intracellular molecular pathways between the lumen and the hepatic portal system, reducing gut permeability, preventing toxins from entering the bloodstream, and causing systemic inflammation (19, 69). Thus, butyrate has been shown to have anti-inflammatory and anti-cancer effects, persuading apoptosis of colon cancer cells and regulating gene expression by histone deacetylase inhibition (5).

Some studies have found that acetate and propionate are associated with weight loss and improved insulin sensitivity, possibly by reducing the absorption of carbohydrates in the gut, so it has been studied as a potential treatment for conditions such as obesity, type 2 diabetes, and certain types of cancer (68). On the other hand, studies have also found that butyrate may have neuroprotective effects and may benefit people with multiple sclerosis and Alzheimer’s disease (70, 71).

Recent research has suggested that SCFAs have a beneficial effect on cardiovascular health. Several studies have found that consuming a diet high in dietary fibers, which promote the production of SCFAs in the gut, is associated with a lower risk of CVD. One of the ways in which SCFAs may protect against CVD is by reducing inflammation, a known risk factor for CVD, and suppressing the inflammatory response. The SCFAs may also help improve lipid metabolism, essential for cardiovascular health (19). Some studies have found that consuming SCFAs can improve lipid profiles, such as lower low-density lipoprotein (LDL) and higher high-density lipoprotein (HDL) levels (72). Chen et al. treated Caco-2 cells with SCFAs to see whether they affected the genes’ expression in cholesterol absorption. Butyrate was shown to inhibit NPC1L1 and to increase ABCG5/G8 gene expression in a dose-dependent manner while increasing the transcriptional activity of liver X receptors in these cells, suggesting that butyrate protects against the development of atherosclerosis (73). Moreover, SCFAs have been found to play a role in regulating glucose metabolism, which is vital for preventing type 2 diabetes, a risk factor for CVD, with studies finding that consuming SCFAs can lead to improvements in insulin sensitivity, lowering blood sugar levels and reduce the risk of developing diabetes (74, 75).

Inhibiting the growth of dangerous pathogens such as *Salmonella* spp. and *Escherichia coli* while promoting the growth of good bacteria like *Lactobacillus* and *Bifidobacteria* are also effects of high concentrations of SCFAs in the gut lumen (72). Additionally, they may help improve the endothelial cells’ function that lines the blood vessels, helping to reduce the risk of atherosclerosis and other cardiovascular problems.

Once SCFAs are absorbed into the bloodstream through the walls of the large intestine by a process known as passive diffusion, they are transported to the liver via the portal vein and then distributed to several tissues, where they can interact with specific receptors such as G protein-coupled receptors (GPRs) and influence gene expression, cellular metabolism and immune response (66, 76). Acetate and butyrate will mainly participate in lipid biosynthesis, and propionate will mainly participate in gluconeogenesis (66).

Overall, SCFAs and gut microbiota are closely interlinked. Maintaining a healthy balance of gut microbiota and sufficient intake of dietary fibers and non-digestible carbohydrates can support the production of SCFAs and help promote overall gut health, positively affecting cardiovascular health. However, more research is needed to fully understand the effects of SCFAs on CVD, and more human studies are needed to confirm the findings.

### 3.2 Trimethylamine N-oxide

**Figure 1:**
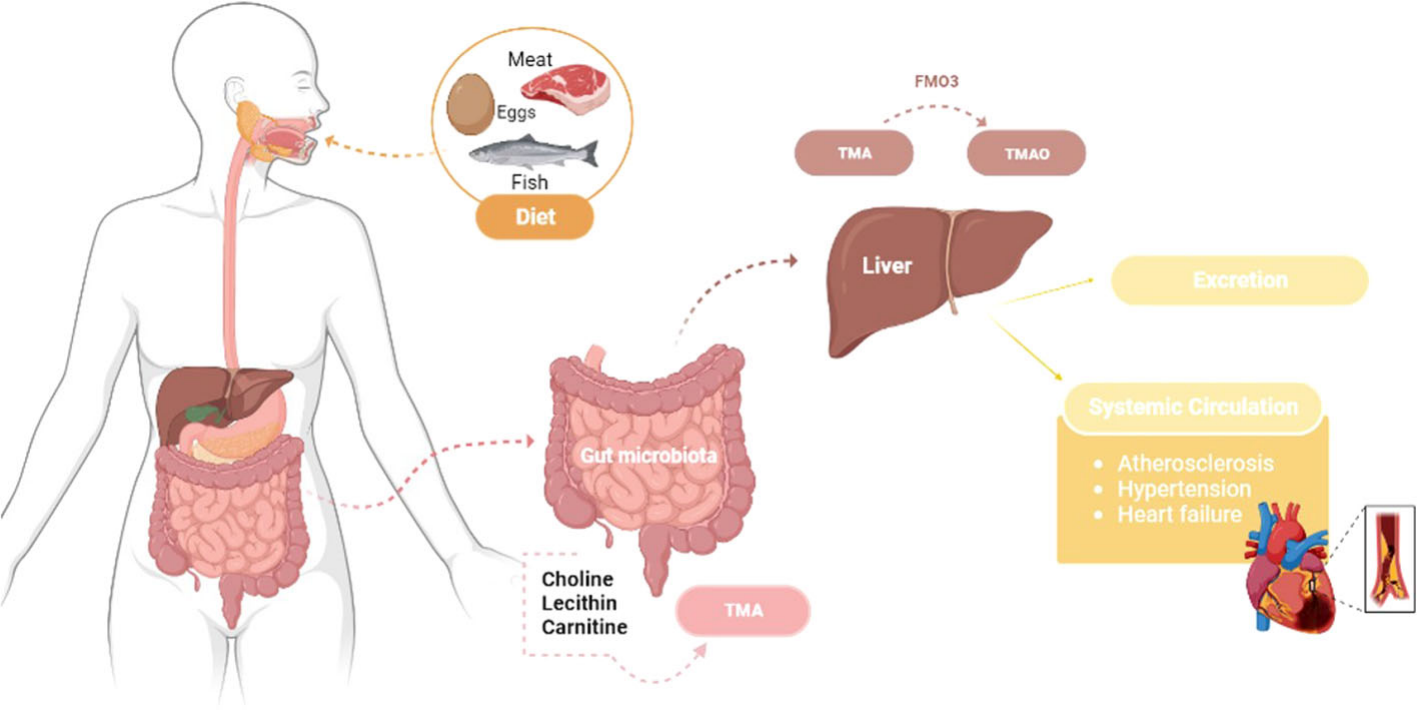
Trimethylamine N-oxide pathway: from food intake to CVDs development – transformation of dietary choline, lecithin, and carnitine into TMAO through gut microbiota metabolism and hepatic oxidation by the hepatic flavin-containing monooxygenase 3 (FMO3), which can be absorbed by extrahepatic tissues or excreted in urine. Atherosclerosis, hypertension and heart failure are all impacts of TMAO that can lead to CVDs. TMA - Trimethylamine; TMAO - Trimethylamine N-oxide.

The gut microbiota plays a crucial role in TMAO formation, as different types of bacteria have different abilities to break down and produce trimethylamine (TMA) and TMAO. Some studies have shown that certain types of bacteria, such as those from the Prevotella and Bacteroides genera, are more efficient at producing TMA and TMAO than others (3, 16). The TMAO is a metabolite produced by certain gut bacteria when they break down foods containing choline, lecithin, and carnitine, commonly found in red meat, eggs, fish, and dairy products. It depends on the initial formation of the TMA compound by the microbiota present, especially in the first portion of the colon, which is absorbed and transported to the liver by the portal circulation, where it is metabolized by hepatic flavin-containing monooxygenase 3 (FMO3) to form TMAO (21). Then, the liver can release TMAO, which will be taken up by extra-hepatic tissues or eliminated by perspiration or urine (**Figure 1**). However, this compound can also be absorbed by macrophages during the formation of atherosclerotic plaque, with TMAO molecules binding to specific receptors on the surface of the macrophages, which triggers a series of signaling events inside the cell that activate specific pathways that induce the expression of genes involved in cholesterol metabolism, inflammation, and oxidative stress (77, 78). This can lead to the accumulation of cholesterol in macrophages and the formation of foam cells, a type of fat-filled cells that can accumulate in the arteries walls and contribute to the development of atherosclerosis (78). In addition, TMAO can regulate the differentiation of monocytes into macrophages and foam cells, influencing pro-fibrotic processes in the heart and kidney through growth factors (79); it can also facilitate the release of calcium ions due to the stimulation of platelet activity, which will activate the prothrombotic pathways (78), and also impairs reverse cholesterol transport, in which the cholesterol is removed from peripheral tissues and transported back to the liver for excretion (80). So, TMAO can play an essential role in regulating inflammation and result in protective or causative effects, stimulating or attenuating the production of inflammatory cytokines that can attract more immune cells, forming a vicious circle that leads to foam cells formation and atherosclerosis development (81).

Some researchers have suggested that TMAO may have a few different functions. It may play a role in regulating gut function, affecting gut motility and modulating the gut barrier function and immune response. Also, it has a role in regulating energy metabolism by modulating the activity of enzymes involved in fatty acid oxidation (82). Nevertheless, TMAO play a role in regulating cardiovascular function, by modulating the function of blood vessels and platelets, which may contribute to the development of CVDs (21). Therefore, studies have shown that high levels of TMAO in the blood may be associated with an increased risk of heart attack and stroke, and high levels of TMAO can cause platelet hyper-responsiveness to various agonists in both humans and animals, which increases vascular inflammation and has a prothrombotic direct effect (21, 83). These effects are likely related to the pathophysiology of type 2 diabetes, obesity, and CVDs (84). Nevertheless, TMAO has also positively affected diabetic peripheral neuropathy, glucose tolerance, and arterial hypertension (85).

The mechanisms by which TMAO promotes atherosclerosis are not fully understood; however, several potential mechanisms have been proposed. One of the most outstanding is related to the activation of NLRP3 inflammasome, a crucial mediator of inflammation, and a significant contributor to the development of atherosclerosis, along with the modulation of the gut microbiota, leading to the production of other harmful metabolites (16, 86). Heianza et al. aimed to evaluate the relationship between gut microbiota metabolites and the risk of major adverse CVD events and death, and after analyzing 19 prospective studies, the authors found that higher levels of TMAO and its precursors were associated with a higher risk of major adverse cardiovascular events and all-cause mortality (87). This may be a potential biomarker for predicting CVD risk, and further research is needed to understand the mechanisms underlying the association between gut microbiota metabolites and CVDs.

Overall, TMAO is recognized as one of the most promising metabolites that may be an independent risk factor for CVDs. A potential therapeutic target for CVDs measuring TMAO levels in blood or urine may help identify individuals at high risk for CVD (59, 88). Research on TMAO and how it contributes to the onset of atherosclerosis is still emerging, but TMAO may be a significant factor in this condition. More research is required to establish the most effective approaches to prevent or treat CVDs and better understand the mechanisms underlying this connection. Therefore, this complex process involves multiple steps and signaling pathways, and understanding this is essential to develop new strategies to prevent or treat atherosclerosis and other related conditions.

### 3.3 Bile acids

Traditionally, bile acids (BAs) were known only for their relevance in lipid metabolism. They are essential molecules produced in the liver and secreted into the small intestine, influencing dietary fats’ breakdown and absorption (89). However, recently, BA has been associated, directly or indirectly, with immune signaling, metabolism, differentiation, and microbiota modulation (90, 91).

The substrate for BAs is cholesterol, and then, through the enterohepatic circulation, these BAs are deposited in the gallbladder. The BAs can be divided into primary or secondary. The most common primary BAs, produced in the liver are cholic acid (CA) and chenodeoxycholic acid (CDCA). After the conjugation with bile salts, glycolic acid (GCA), taurocholic acid (TCA), glycochenodeoxcholic acid (GDCA), taurochenodeoxycholic acid (TDCA), and ursodeoxycholic acid (UDCA) are obtained. The secondary BAs result from the synthesis of the bacterial portion of microbiota, through 7α dihydroxylation, in the small intestine and is deoxycholic acid (DCA) and lithocholic acid (LCA), the latter being the most hydrophobic (24, 91). One of the recently discovered functions of BAs is the fact that they can be used as hormones, mainly for farnesoid X receptor (FXR) and G Protein-coupled membrane receptor 5 (TGR5), to decrease fatty acid oxidation, triglyceride accumulation, and NF-kB inactivation in the aorta (23, 92).

These compounds can affect the diversity of the gut microbiota by altering the growth and survival of certain bacterial species, as they can act as signaling molecules that regulate the expression of genes involved in bacterial metabolism, virulence, and antibiotic resistance, which could result in alterations to the gut microbiota, having both favorable and unfavorable consequences on human health. Recently An et al. demonstrated that depending on the type of microbial strain and particular BA, they can have marked antibacterial effects against the gut microbiota, both *in vitro* and *in vivo*, and according to the findings of this investigation, colonic microorganisms are more vulnerable to BAs than cecal microbes (93). Also, Quinn et al. established the ability of the gut microbiota to conjugate BAs with different molecules like amino acids, producing phenylalanocholic acid, tyrosochilic acid, and lithocholic acid, which are found in humans and enriched in patients with inflammatory bowel disease or cystic fibrosis (94). In addition to their impact on bacterial diversity, BAs can serve as growth substrates for specific bacterial species, including those that produce SCFAs. They can also stimulate the gut epithelium’s production of antimicrobial peptides, which helps protect against pathogenic bacteria (95). Also, BAs can alter the function of the gut barrier by controlling the production of tight junction proteins, which are crucial for preserving intestinal integrity and limiting the translocation of bacteria and toxins across the gut epithelium (96).

Nevertheless, the intestinal microbiota can impact the metabolism of BAs. Some types of gut bacteria can convert primary BAs into secondary BAs, which are different from primary BAs and have other purposes. For instance, studies have demonstrated secondary BAs’ anti-inflammatory and anti-cancer properties (97, 98).

While the specific mechanisms underlying these effects are still being elucidated, it is clear that BAs play a crucial role in maintaining the health of the gut ecosystem, and their interaction is dynamic and complex. In summary, elevated secondary BAs and increased ratios of secondary BAs: primary BAs are more associated with CVD (23, 99). One of the best research pathways to understand the impact of microbiota in cardiovascular disease is focusing on BAs metabolism, particularly secondary BA, its effects, and its relevance in CVD physiopathology (24). Additional research may lead to new therapeutic approaches for the treatment of gut-related disorders like inflammatory bowel disease, obesity, type 2 diabetes, and CVDs, and is essential for developing a comprehensive understanding of human health and disease.

### 3.4 Polyamines – cadaverine, putrescine and spermidine

Cadaverine and putrescine are polyamines synthesized by bacteria. Bacteria often produce them during the decomposition of animal or plant tissue, contributing to unpleasant odors associated with decay and putrefaction. Cadaverine originated from L-lysine through lysine decarboxylase LdcC or acid-inducible CadA (100, 101). Moreover, putrescine arises from synthesizing the substrate L-carnitine, by SpeC or SpeF or the substrate L-arginine, through SpeA and SpeB (102, 103). Spermidine originated from the substrate S-adenosine-L-methionine decarboxylated and putrescine through SpeE (104, 105). Cadaverine and putrescine are later degraded, by the lysine degradation pathway, to succinate (106, 107). Spermidine is only degraded to N-acetylspermidine through SpeG (108, 109). All these polyamines have modulatory effects on the microbiota to promote cardiovascular protection (22, 110).

However, the causal relationship between polyamines and cardiovascular benefits is still in the beginning. Liu S. et al. exploited the effect of one of the polyamines, spermidine, in a mouse model of abdominal aortic aneurysms (AAAs). First, AAAs are associated with a remarkable microbiota dysbiosis, with diminished alpha and beta diversity, accompanied by a shift in bacterial composition, namely increased *Bacteroides* spp., which are pro-inflammatory species, and lower concentrations of *Oscillospira* spp. and *Ruminococcus* spp., species with anti-inflammatory properties. Moreover, the described microbiota dysbiosis upheld functional modifications, especially in polyamines. Furthermore, when spermidine was administered, the intestinal microbiota was modulated with increased concentrations of *Prevotella* and *Desulfovibrionaceae* and decreased wholes of *Parabacteroides* (111). In this study, it was observed that the protective effect of spermidine seems to be associated with a modulation of gut microbiota composition into a more anti-inflammatory one, as well as in the increment of *Desulfovibrionacea* species that can improve polyamine metabolism and promote a more resilient intestinal barrier (111, 112).

Moreover, spermidine is essential for a better heart failure prognosis. This polyamine can act by two different pathways: 1) Direct pathway, where spermidine can avoid cardiac hypertrophy, diminish systolic blood pressure, improve echocardiographic parameters, decrease fibrosis, and, therefore, postpone the progression of heart failure; 2) Indirect pathway, where spermidine can modify intestinal microbiota, decreasing F/B ratio and raising the levels of *Muribaculaceae* spp., therefore ameliorating the intestinal microenvironment *Muribaculaceae* spp. are Gram-negative bacteria found in mice intestines (113), especially after acarbose treatments, since these bacteria produce propionate, a SCFA with anti-inflammatory properties, which is associated with increased longevity in mice (114).

Both cadaverine and putrescine are toxic to humans and animals in large quantities, and they can cause a range of adverse health effects, including nausea, vomiting, and respiratory problems (115). However, polyamines’ relationship with cardiovascular benefits are important since they might have implications for the promotion of improved cardiovascular health.

## 4 Interactions between the gut microbiota and cardiovascular diseases

### 4.1 Atherosclerosis

Atherosclerosis is a chronic inflammatory condition in which the arteries become narrowed and hardened, with an accumulation of lipids and cells, such as white blood cells, endothelial cells, and foam cells in the membranes, resulting in the formation of plaques in the arteries (116, 117). In this condition, innate and acquired immunity are involved, and inflammation of vessel walls is an essential feature of atherosclerosis, contributing to plaque instability and thrombotic occlusion of arteries (118, 119). This process can lead to serious health problems, like heart attacks, strokes, and acute CHD (117).

Recent research has highlighted the potential role of gut microbiota in the development of atherosclerosis by promoting inflammation and altering lipid metabolism (41). In fact, by describing a case of bacterial translocation from the gut to the heart and the discovery of gut bacterial DNA in atherosclerotic plaques, recent studies have established the gut as a potential reservoir of pathogenic microorganisms and with TMAO shown to be involved in the development of the disease (39). One way gut microbiota can promote inflammation is by producing pro-inflammatory compounds such as LPS that can activate immune cells and promote the recruitment of inflammatory cells to the arterial wall (120). Additionally, gut microbiota can also modulate the production of other pro-inflammatory molecules, such as TNF-α, IL-1β, and IL-6, which can contribute to the development of atherosclerosis (120, 121). Another way is by altering lipid metabolism, converting dietary components such as choline, lecithin, and carnitine into TMAO which can increase the uptake of lipids by cells in the blood vessel walls and promote the formation of plaques (122). Moreover, gut microbiota can also affect the host’s insulin resistance and glucose metabolism and the levels of certain hormones such as leptin and ghrelin, which can lead to increased inflammation or regulate appetite, leading to the development of atherosclerosis (123).

This disease develops gradually over time; one of the critical pathways involved in its development is the independent-metabolism pathway, characterized by the accumulation of lipids, particularly cholesterol, in the endothelial cells lining the blood vessels (39, 124). The process begins with injury to the endothelial cells, which can be caused by several factors, such as hypertension, smoking, and diabetes (119). Once the endothelial cells are damaged, they become more permeable, allowing lipids to accumulate in the blood vessels tunica intima, the innermost layer of the arteries. This accumulation triggers an inflammatory response which results in the recruitment of monocytes to the injury site, converting them into foam cells, which are characterized by their high content of lipids, resulting in foam cells and other inflammatory cells, along with extracellular matrix components and smooth muscle cells, to form a plaque on the inner wall of the vessels (124). As the plaque grows, it can block blood flow through the vessel, and if a blood clot forms or a rupture occurs, it can cause serious complications such as heart attack or stroke (125, 126). So, the independent-metabolism pathway is a pivotal contributor to the development of atherosclerosis and its associated complications (121).

The metabolism-dependent pathway is another mechanism that contributes to the development of atherosclerosis. By changing the production of different metabolites, dysbiosis can also have pro-atherosclerotic effects. The TMAO is one of the primary metabolites that play a significant role in atherosclerosis progression, as mentioned above (41, 121, 122). This pathway is also characterized by the accumulation of lipids, particularly triglycerides, in the liver and adipose tissue, and the process begins with the overconsumption of calories and/or a diet high in saturated and trans fats, which leads to an increase in the production of very low-density lipoprotein (VLDL) particles in the liver. These particles are rich in triglycerides and are transported to adipose tissue, where they are taken up by adipocytes and converted into triglyceride-rich lipoproteins (TRLs). Their accumulation in adipose tissue leads to insulin resistance and inflammation, both of which contribute to the development of atherosclerosis as insulin resistance progresses, the adipose tissue secretes higher levels of adipokines, signaling molecules that promote inflammation and increase the risk of atherosclerosis. Additionally, the accumulation of TRLs in the liver produces more extensive and denser LDL particles, which are more prone to sticking to the blood vessel walls and contribute to developing plaques (124). It is important to note that, like the independent-metabolism pathway, the dependent-metabolism pathway is not the only mechanism that contributes to the development of atherosclerosis, and it may act together with multiple pathways to contribute to the disease (122).

It is important to note that the relationship between gut microbiota and atherosclerosis is complex and still not fully understood, so more research is needed to determine the specific mechanisms by which gut bacteria contribute to the development of this disease and how to exploit this information to develop new therapeutic strategies. Therefore, controlling the risk factors, such as maintaining a healthy diet, regular physical activity, and avoiding smoking, can help lower the risk of developing atherosclerosis.

### 4.2 Hypertension

One of the most critical public health issues is hypertension, which increases the risk of pathological strokes, CHD, kidney failure, and early mortality, estimated to affect around one-third of adults worldwide (127). Genome-wide association analyses reveal that only 5% of hypertension occurrence can be explained by genetics, being assumed to be fueled by a combination of genetics and lifestyle variables (128). Environmental elements like dietary salt intake, alcohol use, and inactivity are also linked to increased blood pressure (59, 127).

The exact mechanism by which gut microbiota influence hypertension is not fully understood, but their link has recently been the subject of numerous animal and human studies (129, 130). Hypertension occurrence is often accompanied by gut microbiota imbalance, including decreased diversity, altered enterotype distribution, and variation in bacterial populations, and it is thought that certain types of gut bacteria may produce substances that can affect blood pressure; for example, some bacteria may produce SCFAs that have anti-inflammatory effects, while others may produce substances that increase inflammation and contribute to the development of hypertension (130). Additionally, gut microbiota may influence hypertension by affecting how the body processes and metabolizes nutrients, such as sodium and potassium, given that these nutrients play a crucial role in regulating blood pressure, and an imbalance can lead to high blood pressure (131).

Dysbiosis can accelerate the development of hypertension, described as a slight reduction in the artery lumen that raises peripheral vascular resistance and leads to high blood pressure and atherosclerosis (132). Although the direct connection between hypertension and TMAO has not yet been fully established, it is known that it prolongs the hypertensive effect of angiotensin II and determines an increase of vascular inflammation and a direct prothrombotic effect by the promotion of platelet hyper-responsiveness to multiple agonists both in humans and rodents (133, 134). Blood pressure regulation is generally linked to the renin-angiotensin system, which involves the angiotensin-converting enzyme (ECA) (83). Studies have also found that individuals with higher levels of TMAO in their blood tend to have higher blood pressure compared to those with lower levels, and reducing TMAO levels through dietary interventions, such as decreasing the intake of animal-based protein and fat, has been shown to lower blood pressure in some individuals (84).

To sustain host immunity and gut microbiota homeostasis, SCFAs are essential. Kang et al. demonstrated that SCFAs produced by gut microbiota are involved in modulating blood pressure and can potentially affect the secretion of renin and blood pressure by stimulating host G-protein-coupled receptor (GPR) pathways (135). Yang et al. demonstrated in two rat models that hypertension was associated with gut microbiota dysbiosis, characterized by an increased F/B ratio, a sharp decline in acetate and butyrate-producing bacteria, and an accumulation of lactate-producing bacteria (13). Li et al. demonstrated that hypertension is associated with an increase in the populations of *Klebsiella, Prevotella, Coprobacillus, and Enterobacter* and a decrease in the populations of *Anaerotruncus, Coprococcus, Ruminococcus, Clostridium, Roseburia, Blautia and Bifidobacterium*, correlated with a reduction of F/B ratio and in the production of SCFAs (136). Also, in a review by Verhaar et al. these results were discussed (137). In animal studies, acetate and propionate were also associated with lowering blood pressure and had cardiovascular preventive effects (130).

Animal models of hypertension, such as Dahl-sensitive rats, spontaneously hypertensive rats, angiotensin-II-induced hypertensive rats, and deoxycorticosterone acetate-salted mice, exhibit different gut microbiota compositions from wild-type animals, like a lower abundance of SCFAs-producing bacteria and Bacteroidetes, and higher abundance of lactate-producing bacteria, Proteobacteria and Cyanobacteria (129, 138, 139). Overall, animal models of hypertension help study the disease’s underlying mechanisms and test potential treatments; however, it should be noted that the results obtained from animal models may not always translate to humans.

Therefore, the relationship between gut microbiota and hypertension is complex and not fully understood, but gut microbiota may contribute to developing and managing high blood pressure. Further research is needed to fully understand this relationship and determine the best ways to manipulate the gut microbiota, reduce TMAO levels, and improve cardiovascular health.

## 5 Therapeutical interventions

Therapeutic interventions on gut microbiota use several strategies to manipulate the composition and function of the gut microbiota to improve health. They can include a variety of strategies, such as probiotics, prebiotics, antibiotics, diet, physical activity, and fecal microbiota transplantation (140). These have been used to treat a variety of conditions, and studies have also suggested that gut microbiota modulation could have the potential to not only improve gut health but also reduce the risk of developing CVDs and improve overall health and well-being (6, 141). To restore gut barrier integrity, treatments like probiotics or drugs are probably doomed to failure if used alone. Instead, lifestyle adjustments that consider factors like exercise, sunlight exposure and vitamin D levels, circadian rhythm modulation, and stress management are more likely to produce favorable outcomes (26).

### 5.1 Probiotics, prebiotics, and symbiotics

Some studies suggest that therapeutic interventions aiming at the gut microbiota are effective in treating and preventing CVDs, and they mainly involve probiotics, prebiotics, or symbiotics (142, 143). Probiotics and prebiotics have a critical role in nutrition, sickness, and health, which has boosted their importance in research and commercial circles worldwide. Their use has been studied concerning CVDs, including atherosclerosis, hypertension, diabetes, and metabolic syndrome, with promising results (144–146), as observed in **Table 1**.

**Table 1:** Animal studies and clinical trials using probiotics and prebiotics in several cardiovascular diseases as a therapeutic approach.

Study	Year	Disease	Treatment	Type of study	Via	Outcome	Reference
Sun et al.	2016	Ischemic stroke	*C. butyricum*	No mention	Animal study	Protective effects against ischemic stroke; attenuate neurological deficit, ameliorate histopathological changes alleviate oxidative stress and inhabit apoptosis.	(147)
Tenorio-Jiménez et al.	2018	Metabolic Syndrome	*Lactobacillus reuteri* V3401	5 × 109 CFU/mL	Clinical trial	2-week administration of L. reuteri V3401 in capsules was associated with lower levels of inflammation biomarkers, such as TNF-, IL-6, IL-8, and sICAM-1, and a reduced risk of CVD in obese adults with metabolic syndrome.	(148)
Raygan et al.	2018	Type 2 diabetic patients with Coronary Heart Disease (CHD)	*L. acidophilus, L. reuteri, L. fermentum, Bifidobacterium bifidum* and Selenium	200 μg/day selenium + 8×109 CFU/day probiotic	Clinical trial	Probiotic and selenium co-supplementation reduce inflammatory factors and oxidative damage through producing short chain fatty acids in the gut and the decreasing production of free radicals, and due to blocking activation of nuclear factor-kB through modulating selenoprotein genes expression and inhibiting production of reactive oxygen species.	(149)
Hassan et al.	2020	Atherosclerosis	*Lactobacillus plantarum* ATCC 14917	0.2 mL (109 CFU)	Animal study	L. plantarum ATCC 14917 supplementation decreases the progression of atherosclerotic lesion formation by alleviating the inflammatory process and lowering oxidative stress.	(150)
Mähler et al.	2020	Hypertension	*L. paracasei, L. plantarum, L. acidophilus, and L. delbrueckii; Bifidobacteria longum, B. infantis, and B. breve; Streptococcus thermophilus*	9 × 1011 CFU	Clinical trial	Probiotic can convert dietary components into active metabolites that cause a reduction of pro-inflammatory immune cell function and promote a BP-lowering effect.	(151)
Li et al.	2021	Heart Stroke	*Bacillus licheniformis* CMCC 63516	1 × 108 CFU/mL	Animal study	Preventive effects on heat stroke in rats by sustaining intestinal barrier function, such as increasing tight junctions and decreasing intestinal injury and modulating gut microbiota by increasing the ratio of *Lactobacillus* and *Lactococcus.*	(152)
Wang et al.	2023	Hypertension	*Clostridium butyricum*-pMTL007-GLP-1	109 CFU/mL	Animal study	CB-GLP-1 had markedly reduced blood pressure and improved cardiac marker ACE2, AT2R, AT1R, ANP, BNP, β-MHC, α-SMA and activating AMPK/mTOR/p70S6K/4EBP1 signaling pathway.	(153)

ACE-2 -Angiotensin-converting enzyme type 2; ANR/BNR - Atrial/brain natriuretic receptor; AT1R/2R - angiotensin-II receptor type 1/2; BP - blood pressure; BCKADC - Branched-chain alpha-keto acid dehydrogenase complex; CFU - colony-forming unit; CVD - cardiovascular disease; F/B - Firmicutes-Bacteroides; FMT - Faecal Microbiota Transplant; GLP -1 - Glucagon-lyke peptide type 1; IL - interleukin; MHC - major histocompatibility complex; SCFA - Short chain fatty acids; SMA - spine muscular atrophy; TMAO - Trimethylamine N-oxide; TNF - tumour necrosis factor.

Probiotics are ‘live strains of strictly selected microorganisms which, when administered in adequate amounts, confer a health benefit on the host’, so strictly selected strains can have this potential and only in adequate amounts, as higher doses doesn’t offer the same benefit (154). Probiotics’ positive impact on human health or their ability to prevent disease is mainly brought on by their ability to compete with pathogenic microorganisms, antagonize pathogens, modulate gut microbiota composition, alter pH, or regulate the host’s immune response (146). *Lactobacillus, Bifidobacterium, Lactococcus, Streptococcus*, and *Enterococcus* are among the lactic acid bacteria that make up most of these. Their effects on CVDs are strain-specific and depend on the dose, duration, and specific population studied (144, 145). Several studies have suggested that probiotics can have a beneficial effect on CVDs by reducing inflammatory mediators and blood glucose levels, ameliorating the epithelial barrier function, and competing against pathogens with nutrients and adhesion sites, with some probiotic strains being found to lower blood pressure, and regulating cholesterol levels (26, 145).

Prebiotics are ‘non-digestible food ingredients that beneficially affects the host by selectively stimulating the growth and/or activity of one or a limited number of bacteria in the colon, and thus improves host heath’ (155, 156). Just like probiotics, one way in which prebiotics may be beneficial to CVDs is through their effects on gut microbiota, helping improve gut barrier function and reducing inflammation. Some prebiotics like fructooligosaccharides (FOS) and galactooligosaccharides (GOS) have been found to lower cholesterol levels by reducing the absorption of cholesterol from the gut and by increasing the production of BAs (157). Intestinal enzymes can break down neither oligosaccharides nor polysaccharides. Hence, the gut microbiota transports prebiotics to the colon, where they are fermented, and consequently, their adverse effects are caused mainly by their osmotic properties (155). Besides that, prebiotics are believed to have no severe or potentially fatal adverse effects (144, 158).

Symbiotics are a combination of probiotics and prebiotics; the idea behind it is that by combining the two, the probiotic will have better survival and colonization in the gut, leading to a more significant beneficial effect on the host (158). These have been studied for various health benefits, including improving gut health, boosting the immune system, and reducing the risk of certain diseases such as allergies, obesity, and diabetes. Also, they have been studied for their potential in treating certain gastrointestinal disorders such as inflammatory bowel diseases (IBD) and irritable bowel syndrome (IBS) (159, 160). These supplements could help restore the normal gut microbiota, encourage the growth of good bacteria, and stop the spread of pathogens. By focusing on the gut microbiota and preserving immune homeostasis in the body, probiotics, prebiotics, and symbiotics may be considered promising intervention strategies to prevent or improve CVDs. However, it is essential to note that while health benefits are observed, they should not be relied upon solely to treat or prevent CVD.

Antibiotics are a class of drugs used to treat bacterial infections; however, they can also have unintended consequences on the gut microbiota (161). When antibiotics are taken, they target the pathogenic bacteria causing the infection and the beneficial bacteria that comprise the gut microbiota. The high intake of antibiotics disrupts the delicate balance of the gut microbiota, causing an imbalance and favoring systemic diseases (162). One of the most common effects is diarrhea, caused by the overgrowth of pathogenic bacteria such as *Clostridium difficil*e (163). Antibiotics can also increase the risk of other infections and contribute to the development of antibiotic resistance. Additionally, antibiotics can have long-term effects on the human microbiota. Studies have shown that they can alter the gut microbiota composition for up to a year after the treatment, leading to a decrease in the diversity of bacteria present and an overgrowth of potentially harmful bacteria (162). This disruption could lead to an increase of pro-inflammatory cytokines production, oxidative stress, and impaired endothelial function, which could trigger systemic inflammation, insulin resistance, and endothelial dysfunction, all of which contribute to the pathogenesis of CVDs (164–166). In fact, some studies demonstrated an increased risk of CVDs, such as myocardial infarction and stroke, in patients who received specific classes of antibiotics, like macrolides or fluoroquinolones (167–169). Beyond all, this can lead to other health problems such as allergies, obesity, inflammatory bowel disease, and mental health disorders (170, 171). Therefore, by introducing probiotics and/or prebiotics during or after antibiotic treatments, the balance of the gut microbiota can be restored, through eliminating harmful bacteria and enhancing the gut barrier function, contributing to reduce risk of CVDs and others (172, 173).

Thus, probiotics, prebiotics, and symbiotics all play a role in maintaining digestive and overall health, including cardiovascular health, especially when antibiotics are in question; however, while these supplements can be helpful, they should not replace a balanced diet, exercise, and medical advice in the prevention and treatment of CVDs.

### 5.2 Nutrition and physical activity

Various factors can influence gut microbiota composition, including age, genetics, and lifestyle (141, 174). In addition to probiotics and prebiotics, dietary and lifestyle changes can also be effective in restoring balance to the gut microbiota and improving cardiovascular health, including increasing the intake of fruits, vegetables, and whole grains and reducing the intake of processed and sugar foods (175). Exercise, stress management, and getting enough sleep are essential to maintaining healthy gut microbiota and preventing CVD, even in high-fat diet situations (176, 177).

As mentioned above, gut microbiota composition can be influenced by various factors, including diet, age, genetics, and lifestyle. Diet plays a significant role in shaping the composition and function of the gut microbiota, and its modulation is one way to improve the gut microbiota and promote overall health (175, 178). The types and amounts of nutrients that are consumed can have a direct impact on the growth and survival of different microbial species. A diet high in processed foods, refined sugars, and saturated fats has been linked to an increase in harmful bacteria, like Proteobacteria and *Bacteroides fragilis*, and a decrease in beneficial bacteria in the gut, which can contribute to the development of CVD, such as hypertension, high cholesterol, and obesity by the production of pro-inflammatory compounds. On the other hand, a diet rich in plant-based fiber, fruits, vegetables, and whole grains can help promote the growth of beneficial bacteria in the gut, such as *Bifidobacteria* and *Lactobacilli*, and reduce the risk of these diseases. These bacteria can ferment dietary fibers and produce SCFAs linked to health benefits, like improving gut barrier integrity, increasing mucus production, antimicrobial proteins, and Treg cells, and affecting tight junction assembly (179, 180). According to multiple clinical trials, the Mediterranean diet, rich in fruits, vegetables, and whole grains, which are all good sources of fiber, has been associated with a reduced risk of CVD and other chronic diseases, as it promotes the growth of beneficial bacteria and blood pressure reduction, as well as promotes protective effects on coronary events, strokes, and heart failure (81, 84). Some studies have also shown that certain dietary fats, such as omega-3 fatty acids, can benefit the gut microbiota and improve CVD (181). Additionally, research has shown that different diets can lead to distinct gut microbiota, and some have suggested that switching to another diet can rapidly change its composition (182). For instance, some studies have shown that switching from a Western diet to a Mediterranean diet can rapidly alter the gut microbiota, with beneficial effects attributed to the high proportion of fibers, mono- and poly-unsaturated fatty acids, antioxidants, and polyphenols (183, 184).

Besides diet, physical activity has won much praise for its capacity to control metabolism, insulin sensitivity, weight, and other aspects of health. However, the importance of exercise in controlling the human gut microbiota is becoming increasingly supported by research. Regular physical activity is part of a healthy lifestyle and helps reduce the risk of developing CVD. Exercise can improve cardiovascular health by reducing blood pressure and cholesterol levels, improving blood flow and reducing the risk of blood clots, strengthening the heart muscle and improving its functions, and controlling weight which will reduce the risk of obesity (12, 185). A critical study by Matsumoto et al. discovered that five weeks of exercise training in rats led to an increase in the production of SCFA-butyrate, which is a metabolite from dietary fiber fermentation by bacteria like *Bifidobacteria*, and this shift was also associated with improved endothelial function and a reduction in the development of CVDs (186). In another study, Monda et al. described that even with a high-fat diet, exercise could reduce inflammatory infiltration and protect gut morphology and integrity (176). However, it is essential to note that while exercise can have many beneficial effects, it is not a substitute for a healthy diet.

### 5.3 Fecal microbiota transplantation

The FMT is a medical procedure involving transferring healthy gut bacteria from a donor to a recipient. The idea behind FMT is to restore a healthy balance of gut bacteria in individuals with an imbalance or lack of beneficial bacteria, a condition known as dysbiosis. In individuals with this condition, the balance of gut bacteria is disrupted, leading to a reduction in the diversity and abundance of beneficial bacteria, resulting in a variety of symptoms, such as diarrhea, abdominal pain, and weight loss, as well as an increased risk of developing chronic diseases like inflammatory IBD, Clostridium difficile infection, and metabolic disorders (187). So far, FMT has had a resoundingly positive clinical impact on recurrent *Clostridium difficile* infection. Recently, ulcerative colitis has been extensively studied in other microbiota-related disorders like CVDs (188, 189). This procedure is typically performed by administering a stool sample from a healthy donor, usually via a colonoscopy, sigmoidoscopy, enema, or orally, to the recipient, aiming to repopulate the recipient’s gut with a diverse and balanced community of bacteria that can improve the overall health of the gut microbiota, and in turn, improve the overall health of the individual (190, 191).

Despite the evidence surrounding CVDs and gut microbiota, few studies have explored the potential effect of FMT on these diseases. Hu et al. centered on the question of whether FMT could be helpful in myocarditis treatment, with a murine model of experimental autoimmune myocarditis, resulting in reduced inflammatory infiltration, improved functions of the blood vessels, and gut microbiota rebalance, proposing a potential therapeutical strategy (192). In another study, Toral et al. demonstrated that transplanting healthy feces into spontaneously hypertensive rats reduces blood pressure by modifying sympathetic nerve activity associated with increased levels of SCFAs (193). Kim et al. also studied FMT impact on CVDs, observing that when hypertensive donors’ feces were transferred to germ-free mice, the recipient mice’s blood pressure rose compared to germ-free mice that received healthy FMT (194). A recent study by Hatahet et al. demonstrated that gut microbiota modulation with FMT associated with butyrate treatment, could alleviate systolic and diastolic function in obese mice (195). On the other hand, Gregory et al. discussed the transmission of atherosclerosis susceptibility using FMT in an animal model, proving that not only positive effects can come from FMT procedure (196). In **Table 2** we resume the findings of some animal studies and clinical trials from the last years.

**Table 2:** Fecal microbiota transplantation results in animal studies and clinical trials in various cardiovascular diseases.

Study	Year	Disease	Treatment	Type of study	Via	Outcome	Reference
Hu et al.	2019	Myocarditis	FMT	Animal study	Oral gavage	Reduced inflammatory infiltration, improved functions of the blood vessels, and gut microbiota rebalance with an increase in microbial richness and diversity. Increase F/B ratio.	(192)
Toral et al.	2019	Hypertension	FMT	Animal study	Oral gavage	Reduced blood pressure by modifying sympathetic nerve activity associated with increased levels of SCFAs.	(193)
Kim et al.	2017	Hypertension and Myocarditis	FMT	Animal study	Oral gavage	Hypertensive donors’ feces were transferred to germ-free mice and the recipient mice’s blood pressure rose compared to germ-free mice that received healthy FMT. Also, obese mice receiving FMTs from healthy resveratrol-fed mice have improved glucose homeostasis, and decreased inflammation and myocarditis	(194)
Gregory et al.	2015	Atherosclerosis	FMT	Animal study	Oral gavage	Atherosclerosis susceptibility was transmitted with FMT	(196)
Hatahet et al.	2023	Heart failure	FMT	Animal study	Oral gavage	Improvement systolic and diastolic early dysfunction following FMT. Both FMT and butyrate plays a significant role in reducing the level of inactive p-BCKDH in the heart.	(195)
Smits et al.	2018	Metabolic Syndrome	FMT	Randomized Controlled Trial	Nasoduodenal infusion	Single lean vegan-donor FMT in metabolic syndrome patients resulted in detectable changes in intestinal microbiota composition but failed to elicit changes in TMAO production capacity or parameters related to vascular inflammation.	(197)

Altogether, these findings point to a significant role of the gut microbiota in the development of CVDs; nevertheless, more human data and clinical trials are required to support the use of FMT in CVD before it can be applied broadly.

While some researchers considered FMT as a safe and effective treatment option for various conditions, with success rates that are often higher than traditional medical treatments, others are still suspicious of the procedure’s benefits. Therefore, it’s still considered an experimental treatment and not yet widely available or approved by regulatory agencies worldwide (187, 198).

## 6 Conclusions

In conclusion, the gut microbiota is a complex and dynamic community of microorganisms that plays a critical role in human health and disease. Emerging evidence suggests that the gut microbiota may be linked to the development of CVD, such as atherosclerosis, hypertension, diabetes, and others. Recent studies have highlighted the importance of the gut-heart axis in the pathogenesis of CVDs, with an increasing body of evidence linking gut dysbiosis its development. Despite the promising results from animal models and some human studies, further research is needed to better understand the mechanisms by which gut microbiota influence the cardiovascular system and to determine the safety and efficacy of these interventions in clinical settings. The potential prophylactic and therapeutic implications of this research are exciting and we look forward to continued advancement of scientific knowledge in this field.

## Author contributions

Conceptualization: CA, JN, and PB, Writing – original draft preparation: CA and JN. Writing – review and editing: CA, JN, DC and PB. Supervision: DC and PB. All authors have read and agreed to the published version of the manuscript.
